# Effect of chronic high-altitude exposure on postoperative pulmonary complications: a retrospective cohort study

**DOI:** 10.1080/07853890.2026.2627063

**Published:** 2026-02-16

**Authors:** Zhang Jianjun, Su Peng, Feng Tianhang, Zhu Zexuan, Lei Qian, Xu Guangmin

**Affiliations:** ^a^ Department of Anesthesia Surgery Center, Sichuan Provincial People’s Hospital, School of Medicine, University of Electronic Science and Technology of China, Chengdu, China; ^b^ Imaging Department, Chengdu Traditional Chinese Medicine Hospital of Pidu District, Chengdu, China

**Keywords:** postoperative pulmonary complications, high-altitude exposure, hypoxia

## Abstract

**Background:**

Many factors can influence the occurrence of postoperative pulmonary complications (PPCs) in the perioperative period, but it is unclear whether chronic high-altitude exposure (CHAE) affects the occurrence of PPCs.

**Methods:**

This retrospective study included 235,128 surgical patients aged 18 years and older from January 2013 to December 2022. The occurrence of PPCs, such as pneumonia, atelectasis, and respiratory failure, was determined based on the admission and discharge diagnoses. To reduce the confounding effects caused by imbalances in demographic and clinical characteristics at baseline, we employed a 1:1 propensity score matching (PSM) to match the CHAE and non chronically high-altitude exposed (NCHAE) patients. Statistical analyses were conducted from January 1, 2025, to March 1, 2025.

**Results:**

A total of 235,128 cases were included, with 11,075 (4.7%) patients experiencing PPCs. There were 8,565 patients with CHAE, of whom 484 (5.7%) developed PPCs. In contrast, there were 226,562 patients NCHAE, with 10,591 (4.7%) experiencing PPCs. After 1:1 PSM, 8,564 CHAE were matched with 8,564 NCHAE. In the CHAE group, 484 (5.7%) experienced PPCs, while 394 (4.6%) in the NCHAE group shoewd a statistically significant difference (*p* = 0.002). Adjusted multivariable conditional logistic regression analysis indicated that CHAE increased the incidence of PPCs (odds ratio [OR], 1.25; 95% CI, 1.02–1.53). Furthermore, the length of hospitalization and postoperative hospitalization duration of patients in the CHAE group were longer than those in the NCHAE group.

**Conclusions:**

This retrospective study suggests an association between CHAE and PPCs within 30 days after surgery. However, the undefined exposure duration highlight the need for prospective studies to definitively establish causality.

## Introduction

In recent years, with advancements in surgical techniques and perioperative management, the incidence of PPCs has decreased; however, PPCs remain one of the most common postoperative complications [1]. They can prolong hospital stays and increase perioperative morbidity and mortality [2]. Over 80 million people live permanently at altitudes above 2,500 meters [3,4], yet we currently lack clarity regarding the occurrence of PPCs in this large population. Similarly, we are also unsure of the impact of prolonged preoperative high-altitude exposure on PPCs.

Adaptive changes occur when the human body enters a high-altitude environment exceeding 2,500 m. The lungs are one of the primary organs involved in acclimatization to high altitudes. Acute exposure to high-altitude environments often results in acute lung injury, a common high-altitude response [5]. Prolonged exposure to high-altitude environments leads to structural and functional adaptations in lungs. In terms of pulmonary function, the decreased partial pressure of oxygen in high-altitude environments prompts hypoxic ventilatory responses and hyperventilation to ensure adequate oxygen supply [6]. After prolonged exposure, the extent of hyperventilation decreases compared to acute exposure, yet hyperventilation still persists [7]. The peak expiratory flow rate in populations residing at high altitudes is increased compared to those at lower altitudes, while forced vital capacity (FVC) decreases [8–10], and forced expiratory volume in one second (FEV1) remains unchanged or slightly increases, resulting in an increased FEV1/FVC ratio [11]. Additionally, residual volume (RV) [12,13], total lung capacity [14], and tidal volume are also elevated in high-altitude populations compared to those at lower altitudes. Prolonged exposure reduces the sensitivity of peripheral chemoreceptors, leading to hypoxic ventilatory acclimatization (HVR), which in turn decreases hyperventilation. Simultaneously, it utilizes various oxygen transport mechanisms to effectively reduce the workload on the respiratory muscles and prevent muscle fatigue. In contrast, to adapt to the hypoxic environment at high altitudes, the lung diffusion capacity is also adaptively enhanced. Structurally, adaptive changes in lung function at high altitudes lead to an increase in the thoracic diameter. Additionally, Chronic hypoxia triggers a series of pulmonary adaptations, including persistent vasoconstriction [15], vascular remodeling [16], elevated pulmonary arterial pressure [17], increased vascular permeability, and reduced pulmonary surfactant due to hypoxia and inflammation [18].

Long-term exposure to low oxygen levels, low pressure, and strong ultraviolet radiation in high-altitude environments increases the incidence of pulmonary disease. The presence of pulmonary disease before surgery is a risk factor for PPCs [19,20]. Furthermore, when individuals who have lived at high altitudes for an extended period temporarily descend to low altitudes, their overall arterial blood oxygen partial pressure remains lower than that of individuals at sea level, and preoperative low arterial blood oxygen partial pressure is negatively correlated with PPCs [21–23].

Therefore, we conducted this retrospective study to assess the impact of CHAE on PPCs within 30 days after surgery. Given the effects of high-altitude exposure on the lungs, we hypothesized that CHAE would increase the occurrence of PPCs in patients.

## Methods

### Ethics

This single-centre retrospective cohort study was approved by the Medical Ethics Committee of Sichuan Academy of Medical Sciences and Sichuan Provincial People’s Hospital (Approval No. 2024-795). The procedures used in this study adhere to the tenets of the Declaration of Helsinki. Given the retrospective nature of this study, the committee waived the requirement for informed consent. This study adheres to the STROBE statement.

### Data collection

We extracted relevant information from the electronic medical record system of the hospital for all patients aged ≥ 18 years who underwent surgical procedures under general anesthesia between January 2013 and December 2022. This included data on their residence address, registered address, age, sex, height, weight, preoperative albumin (Alb), preoperative hemoglobin (Hb), admission and discharge diagnoses, whether the surgery was an emergency (type of surgery, TOS), name of the surgery, duration of the surgery (DOS), whether blood transfusion (BT) was administered during the procedure, fluid supplementation during the procedure, ASA classification, and length of hospital stay.

The inclusion criteria were as follows: 1. patients aged 18 years or older, 2. Undergoing general anesthesia intubation Exclusion criteria: 1. presence of acute or chronic pulmonary infections, chronic obstructive pulmonary disease, pulmonary edema, atelectasis, asthma, bronchiectasis, pleural effusion, or bronchopleural fistula prior to surgery; 2. missing important information; 3. Discrepancies in altitude between residence and registered domicile, such that one is above 3,000 m and the other is below 3,000 m; 4. postoperative hospitalization exceeding 30 days, and 5. Preoperative acute heart failure or acute renal injury.

TOS are categorized into thoracic surgery, abdominal surgery, cardiovascular surgery, orthopedic surgery, gynecological-obstetrical surgery (OB-GYN), neurosurgery, general surgery excluding abdominal surgery (GEA), maxillofacial surgery, urological surgery, and eye, ear, nose, and throat surgery (EENT).

Patients were categorized into CHAE and NCHAE groups based on their current residential and household registration address. Specifically, patients whose current residential and household registration address altitudes both exceeded 3,000 m were classified as the CHAE group, while those whose altitudes were both below 3,000 m were classified as the NCHAE group. A limitation of this method is that it does not allow for determining the duration of residence, creating a potential for misclassification when only address information is used.

The primary outcome of this study was defined as the incidence of PPCs. Patients with PPCs were identified using established diagnostic criteria based on codes from the International Classification of Diseases, Ninth and Tenth Revisions, Clinical Modification (ICD-9-CM and ICD-10-CM). Secondary outcomes included total length of hospital stay and postoperative length of stay.

We defined the incidence of pulmonary complications within 30 days post-surgery as the primary outcome, which included pneumonia, atelectasis, and respiratory failure—respiratory diseases that share a common pathophysiological mechanism [24]. To identify patients with PPCs, we utilized previously published standards based on the International Classification of Diseases - Clinical Modification, 10th Edition diagnostic codes [25]. The secondary outcomes included total length of hospital stay and postoperative length of stay.

### Managing missing data

If the missing values of a variable exceeded 20%, the variable was excluded. The imputation assumed that continuous data followed a multivariate normal distribution, with continuous variables imputed by predictive mean matching and binary variables by logistic regression [26,27]. Simultaneously, we will incorporate all variables related to the missing variables into the archival model to assist in predicting the missing values.

### Statistical analysis

Data analysis was conducted using the R 4.2 statistical software. Continuous data are presented as mean ± standard deviation, and intergroup comparisons were performed using the t-test; for data not conforming to a normal distribution, the rank-sum test was used. Categorical data are expressed as counts and percentages, and group comparisons were performed using the chi-square test. To calculate the odds ratio (OR) and 95% confidence interval (CI) of each independent variable concerning PPCs, variables with a *p*-value <0.2 from univariate analysis were included in multivariate logistic regression analysis. To reduce the confounding effects caused by imbalances in demographic and clinical characteristics at baseline, we employed a 1:1 PSM to match the CHAE and NCHAE patients. The matching included variables with a *p*-value <0.2 from univariate analysis. The matching factors included age, sex, ASA classification, hypertension, diabetes, congestive heart failure (CHF), renal failure (RF), anemia, Alb, TOS, emergency surgery, intraoperative transfusion, and DOS. The PSM was performed using the nearest-neighbor method with a 1:1 ratio and a caliper width of 0.25. A standardized mean difference (SMD) <0.1 indicated balance between groups. Subsequent univariate and multivariate conditional logistic regression analyses were conducted on the matched cohort to evaluate the effect of CHAE on PPCs. The odds ratio (OR) and 95% confidence interval (CI) were used to assess risk ratios. Statistical significance was set at *p* < 0.05.

### Sensitivity analyses

Sensitivity analysis of the primary results was conducted using multiple statistical strategies (see supplementary data S2). 1. Redefinition of exposure: The CHAE group comprised individuals with both their registered and current residences at ≥ 2,500 m above sea level. In contrast, the NCHAE group comprised those with both residences at < 2,500 m. All other inclusion and exclusion criteria remained consistent with the main analysis. 2. A 15-day postoperative observational period was defined for the primary outcome. The definitions for CHAE and all other inclusion and exclusion criteria remained consistent with those used in the main analysis. 3. Continuous variables, including age, duration of surgery, and intraoperative fluid administration, were categorized for analysis. All other study criteria—including inclusion and exclusion criteria as well as the definition of CHAE—remained consistent with those used in the main analysis.

## Results

### Patient characteristics

**Figure 1.**
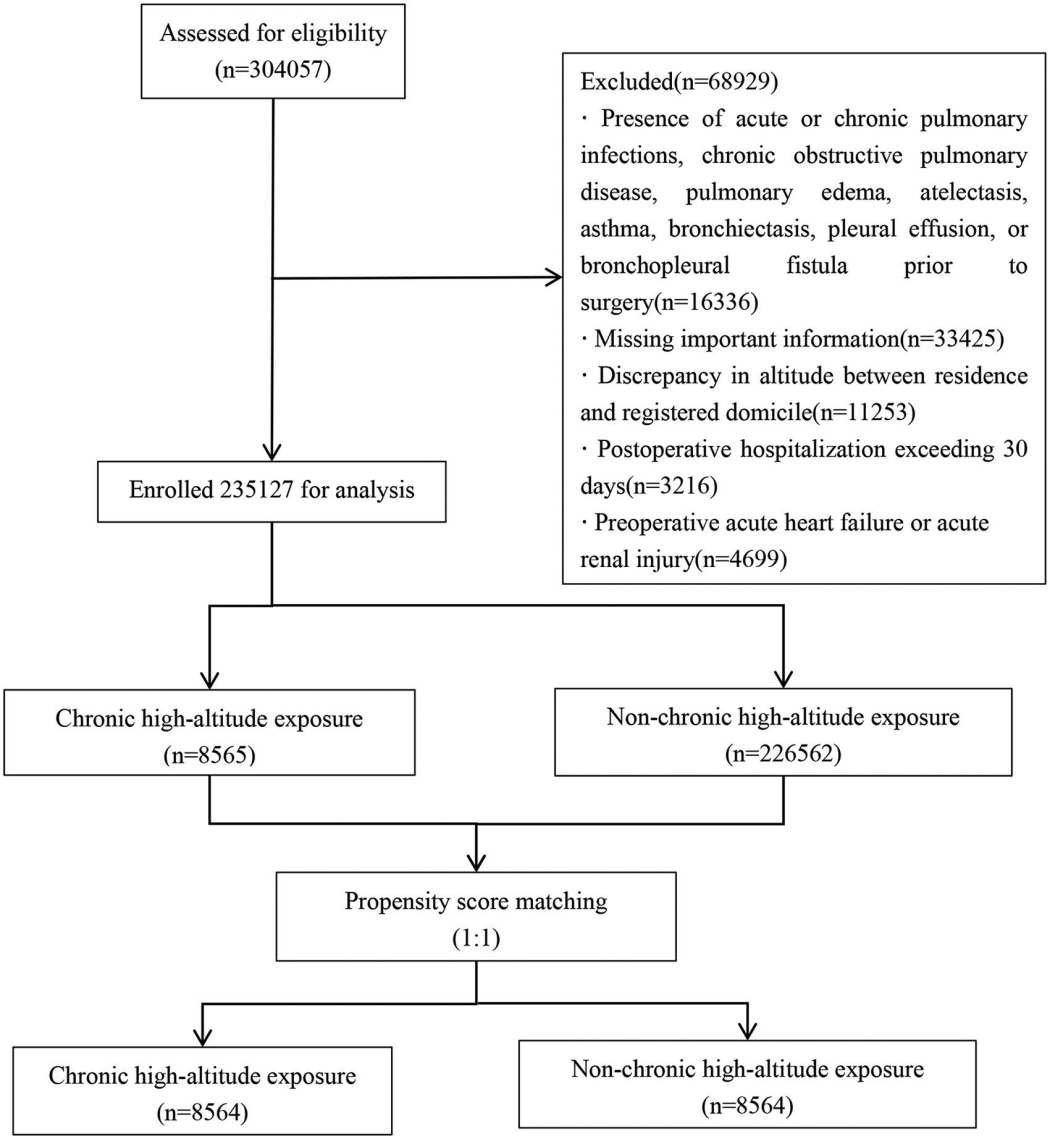
Flow chart of the study.

A total of 304,057 patients were extracted, among whom 16,336 patients had preoperative acute and chronic pulmonary infections, chronic obstructive pulmonary disease, pulmonary edema, atelectasis, asthma, bronchiectasis, pleural effusion, and bronchial fistula. In addition, 33,425 patients had incomplete data. In total, 4,699 patients had preoperative acute renal impairment and acute heart failure. A total of 11,253 patients lived at inconsistent altitudes. Postoperative hospitalization exceeded 30 days in 3,216 patients. In total, data were obtained from 235,127patients (Figure 1). Significant differences were found between CHAE and NCHAE patients in terms of age, BMI, altitude, surgical site, diabetes, hypertension, BT, DOS, and intraoperative fluid administration (Table S1). In the univariate regression analysis, the variables of sex, age, BMI, ASA classification, TOS, emergency, preoperative CHF, RF, hypertension, diabetes, preoperative Alb level, Hb level, intraoperative BT, and DOS had *p*-values < 0.2 (Table S2). These variables were analyzed using PSM, resulting in the successful matching of 8,564 CHAE and 8,564 NCHAE patients. After PSM, the SMD values for all variables were less than 0.1 (Table 1, see detailed information in Table S3). The plots of propensity score distribution and SMDs pre- and post-matching are provided in Supplementary Figure S1 and Figure S2.

**Table 1.** The standard mean difference before and after propensity score matching.

Variable	SMD before PSM	SMD after PSM
Age, year, Median (IQR)	**0.232**	0
Gender, *n* (%)		
Male	0.098	0.031
Female	0.098	0.031
BMI, *n* (%)		
18.5-27	0.065	0.019
<18.5	0.056	0.01
>27	**0.101**	0.015
ASA, *n* (%)		
I	0.091	0.005
II	0.071	0.028
III	0.002	0.046
IV	0.014	0.004
V	0.011	0.005
VI	0.002	
Hb, g/L		
Male:≥130; Female:≥120	0.031	0.022
Male:110–129; Female:110-119	**0.156**	0.033
80–109	0.073	0.001
<80	0.072	0.006
Alb, g/L, *n* (%)		
≥35	**0.224**	0.03
25–35	**0.206**	0.028
<25	**0.083**	0.009
Hypertention, *n* (%)		
No	**0.123**	0.042
Yes	**0.123**	0.042
Diabetes, *n* (%)		
No	**0.208**	0.007
Yes	**0.208**	0.007
CHF, *n* (%)		
No	0.019	0.016
Yes	0.019	0.016
RF (%)		
No	0.027	0.015
Yes	0.027	0.015
Emergency surgery, *n* (%)		
No	0.042	0.006
Yes	0.042	0.006
DOS, min, Median (IQR)	**0.223**	0.018
Blood transfusion, *n* (%)		
No	**0.125**	0.023
Yes	**0.125**	0.023
TOS, *n* (%)		
Thoracic	**0.238**	0.003
Abdominal	0.078	0.022
Cardiovascular	0.033	0.005
Orthopaedic	**0.183**	0.033
OB-GYN	0.065	0.004
Neurosurgical	0.079	0.008
GEA	**0.111**	0
Maxillofacial	0.045	0.017
Urologic	0.059	0.007
EENT	0.035	0.005

IQR, interquartile range; PSM, Propensity Score Matching; ASA, American Society of Anesthesiologists; BMI, body mass index; Alb, albumin; CHAE, Chronic high-altitude exposure; NCHAE, Non-Chronic high-altitude exposure; DOS, Duration of surgery; CHF, congestive heart failure; RF, renal failure; Hb, hemoglobin; TOS, type of surgery; OB-GYN, obstetrics and gynecology; GEA, general except Abdominal; EENT, eyes, ears, nose, and throat surgery; SMD, standard mean difference.

### Primary and secondary outcomes

A total of 11,075 patients developed PPCs within 30 days post-surgery. Among them, 10,714 had postoperative pneumonia, 238 had atelectasis, and 737 had respiratory failure. The overall incidence of PPCs was 4.7%, with patients exposed to chronic high altitude having a rate of 5.7%, while the incidence in NCHAE patients was 4.7%, indicating a statistically significant difference (*p* < 0.001). After PSM, the CHAE group and 484 (5.7%) patients experienced PPCs, while 394 (4.6%) in the NCHAE group showed a statistically significant difference (*p* = 0.002). The incidence of postoperative pulmonary complications also varied by type of surgery, with detailed information available in Table 2. Higher PPC incidence in CHAE across surgical categories may reflect residual confounding or mixed exposure status (acute vs. chronic).

**Table 2.** Incidence of postoperative pulmonary complications in chronic high-altitude exposure and non-chronic high-altitude exposure patients across various types of surgeries, as well as the proportion of each complication.

	Before PSM		After PSM	
TOS	CHAE (*n* = 8565)	NCHAE (*n* = 226562)	CHAE (*n* = 8564)	NCHAE (*n* = 8564)
Overall	484 (5.7%)	10591 (4.7%)	484 (5.7%)	394 (4.6%)
Thoracic	32 (12.8%)	2269 (14.5%)	32 (12.8%)	29 (11.4%)
Abdominal	142 (5.9%)	2427 (4.3%)	142 (5.9%)	119 (5.1%)
Cardiovascular	51 (27.1%)	1260 (32.5%)	51 (27.1%)	48 (24.7%)
Orthopaedic	53 (3.7%)	642 (2.8%)	53 (3.7%)	36 (2.3%)
OB-GYN	11 (0.8%)	186 (0.4%)	11 (0.8%)	5 (0.3%)
Neurosurgical	149(23.8%)	2447 (20.6%)	149 (23.8%)	121 (19.9%)
GEA	19 (2.0%)	517 (1.6%)	19 (2.0%)	12 (1.3%)
Maxillofacial	4 (2.2%)	38 (0.6%)	4 (2.2%)	2 (1.2%)
Urologic	22 (3.4%)	778 (3.8%)	22 (3.4%)	20 (3.2%)
EENT	1 (0.2%)	27 (0.2%)	1 (0.2%)	2 (0.5%)
pneumonia	466 (96.3%)	10248 (96.8%)	466 (96.3%)	375 (95.2%)
respiratory failure	43(8.9%)	694 (6.6%)	43 (8.9%)	32 (8.1%)
atelectasis	5 (1.0%)	233 (2.2%)	5 (1.0%)	10 (2.5%)

PSM, Propensity Score Matching; CHAE, Chronic high-altitude exposure; NCHAE, Non-Chronic high-altitude exposure; TOS, type of surgery; OB-GYN, obstetrics and gynecology; GEA, general except Abdominal; EENT, eyes, ears, nose, and throat surgery.

The postoperative hospitalization duration of patients in the CHAE group was significantly longer than that of patients in the NCHAE group. For further details, please refer to the (supplementary material Table S4 in Supplemental Material Data S1).

### The relationship between CHAE and PPCs

Before matching, a crude analysis (OR, 1.22; 95% CI, 1.11–1.34) and multivariable regression analysis (OR, 1.28; 95% CI, 1.15–1.42) suggested that CHAE was associated with an increased risk of PPCs. After PSM, the adjusted main analysis results (OR, 1.25; 95% CI, 1.02–1.53) also indicated that CHAE was correlated with an increase in PPCs. Please see Table 3.

**Table 3.** Association between chronic high-altitude exposure and postoperative pulmonary complications in the crude analysis, multivariable analysis, and propensity score matching analyses.

Analysis	OR (95 % CI)	*p*-value
Crude analysis	1.22 (1.11–1.34)	<0.001
Multivariable analysis	1.28 (1.15–1.42)	<0.001
PSM		
With matching	1.25 (1.09–1.44)	0.002
Adjusted for PSM	1.25 (1.02–1.53)	0.03

OR, odds ratio; PSM, propensity score matching; CI, confidence interval.

### Sensitivity analyses

Sensitivity analysis indicated that the robustness of the CHAE increases the PPCs. (See Supplementary Material Data S2 for details). After including patients living at 2500–3000 meters, the primary analysis after PSM (For detailed information before and after PSM, please refer to the Table S5 in Supplemental Material Data S2) suggests that CHAE is associated with an increased incidence of PPCs (OR 1.22, 95% CI, 1.08–1.38) (Table S6 in Supplemental Material Data S2). When adjusting the included patients to those discharged within 15 days post-surgery, the primary analysis after PSM (For detailed information before and after PSM, please refer to the Table S7 in Supplemental Material Data S2) also indicated that CHAE was correlated with an increase in PPCs (OR 1.33, 95% CI, 1.10–1.61) (Table S8 in Supplemental Material Data S2). Furthermore, when continuous variables were adjusted for categorical variables, the primary analysis after PSM (For detailed information before and after PSM, please refer to the Table S9 in Supplemental Material Data S2) similarly revealed a relationship between CHAE and an increased occurrence of PPCs (OR 1.21, CI 1.02–1.44) (Table S10 in Supplemental Material Data S2).

## Discussion

Our retrospective study found that the overall incidence of PPCs in patients with CHAE was higher than that in those without CHAE. A history of CHAE was a risk factor for PPCs (OR, 1.25; 95% CI, 1.02–1.53). Additionally, the length of hospital stay for surgical patients with CHAE was longer than that for patients with NCHAE. Sensitivity analyses confirmed the robustness of the results.

Our findings indicate that the overall incidence of PPCs was significantly higher in patients with chronic high-altitude exposure (CHAE) than in non-exposed (NCHAE) patients (5.7 vs. 4.7%, *p* < 0.001). Given the significant baseline differences in demographics, comorbidities, and surgical factors between the groups, we performed an adjusted analysis controlling for key confounders including age, sex, ASA class, BMI, preoperative albumin and hemoglobin levels, congestive heart failure, renal failure, emergency surgery, and intraoperative blood transfusion. Following this adjustment, the incidence of PPCs in the CHAE group remained significantly elevated at 5.7%, compared to 4.6% in the NCHAE group (*p* = 0.002).

The pathophysiological link between chronic high-altitude exposure and PPCs involves a cascade of events. Prolonged hypoxia triggers the upregulation of key factors including HIF-1α, NF-κB, and pro-inflammatory cytokines (e.g. TNF-α, IL-6, IL-8, IL-1β, IL-1α) [28–32], alongside a decrease in alveolar surfactant [18,33], a rise in pulmonary arterial pressure [34], and increased vascular permeability [35–37]. These specific changes—elevated inflammatory markers [38–41], pulmonary hypertension [42,43], reduced surfactant [44,45], and heightened vascular permeability [46–48], are each independently recognized as mechanisms that collectively contribute to the pathogenesis of PPCs.

Few studies have reported the occurrence of PPCs in patients with CHAE. A study involving patients exposed to high altitudes undergoing non-cardiothoracic surgeries lasting more than one hour revealed that the incidence of PPCs varied depending on the PEEP used during surgery. The low PEEP group (PEEP < 5 cmH_2_0) had a PPC rate of 17.1%, while the high PEEP group (PEEP ≥ 5 cmH20) had a rate of 15.8% [49]. However, it should be noted that this study did not include a control group of non-high-altitude patients. Interestingly, another study [50] in patients undergoing liver resection suggested that high-altitude exposure might be associated with a reduced incidence of PPCs. Several factors may explain this discrepancy. First, the definition of “high-altitude” differed between the studies: the cited study used a threshold of ≥1,500 meters, whereas our study defined chronic high-altitude exposure as residence at ≥3,000 meters. This suggests that the impact of altitude exposure on PPC risk may vary with the level and duration of exposure, highlighting the complexity of this relationship. Second, the sample size in the liver resection study was relatively small, which may limit the reliability of its findings.

Similar to patients with NCHAE, the incidence of PPCs in patients with CHAE varies among different types of surgeries. The rates of such complications were higher following major cardiac vascular surgery, neurosurgery, and thoracic surgery, specifically documented at 27.1, 23.8, and 12.8%, respectively. We noted that the incidence of postoperative pulmonary complications reported in our study is lower than that in other research [1,49,51,52], which we attribute to several factors. In our study, a significant number of surgeries had a duration of less than one hour; for instance, 40.4% of patients undergoing abdominal surgery had a surgical duration of less than one hour; Second. Postoperative pulmonary complications that we defined did not include pleural effusion, pneumothorax, pulmonary edema, aspiration pneumonia, bronchospasm, or pulmonary embolism. We assessed whether the patients experienced pulmonary complications based on their admission and discharge diagnoses, which depended on the clinical practices of the treating physicians.

Existing protective ventilation strategies for non-high-altitude populations can effectively reduce the occurrence of PPCs [53,54], and these strategies recommend the use of low tidal volumes and PEEP [20,55]. However, for patients who are chronically exposed to high altitudes, due to increased tidal volumes and functional residual capacity, it remains unclear whether these strategies are suitable for this population. Additionally, personalized ventilation strategies have not been effectively implemented in such patients in clinical practice. A review of previous studies indicates that an end-expiratory positive pressure of ≥5 cm H2O during mechanical ventilation may reduce postoperative pulmonary complications in high-altitude patients [49]. However, there are no related studies on how to set the tidal volume. In our study, we were unable to distinguish between the ventilation strategies used by patients during surgery.

The observed longer hospital stays among CHAE patients may be attributed to several factors. One potential explanation is the geographical barrier to timely healthcare in remote high-altitude regions, which can delay treatment and slow recovery [56]. Additionally, surgery-induced stress may compromise the compensated physiological adaptations (e.g. in blood pressure and renal function) developed at high altitude, leading to more complex postoperative management and extended hospitalization [57–59].

### Limitations

This study is subject to several important limitations that must be considered when interpreting its findings. First, as a single-center, retrospective cohort study, its design carries inherent constraints that affect the validity and generalizability of the results. The non-randomized nature of the data collection introduces the potential for unmeasured confounding and selection bias. Second, the identification of PPCs relied solely on admission and discharge diagnostic codes. This method is susceptible to variability and under-documentation based on clinical practice patterns, which may have led to both misclassification and an underestimation of the true PPCs incidence. Third, patients with postoperative hospital stays exceeding 30 days were excluded from the analysis. Consequently, the incidence and profile of PPCs in this potentially high-risk subgroup remain unknown, limiting the comprehensiveness of our findings. Fourth, exposure classification was based on patient address data, which is an imprecise proxy for the duration and continuity of high-altitude exposure. This likely resulted in exposure misclassification, diluting the observed effect sizes and compromising the accuracy of our risk estimates. Fifth, our PSM was performed only for the diagnoses of hypertension and diabetes. While this adjusted for these key comorbidities, residual confounding likely persists due to imbalances in other unmatched clinical factors (e.g. cardiac function, pulmonary comorbidities), which could bias the estimated association. Finally, several potentially significant confounding variables were not measured or adjusted for, including detailed smoking history, preoperative oxygen partial pressure, and specific intraoperative management parameters (e.g. ventilation strategies, fluid balance). Their omission restricts our ability to establish a fully adjusted causal relationship.

## Conclusions

Our findings suggest an association between CHAE and increased PPCs, given that CHAE may affect the risk of PPCs, a comprehensive strategy should be adopted for individuals living at high altitudes, involving strengthened preoperative risk assessment to identify high-risk patients in advance, formulate personalized treatment plans, reduce the impact of postoperative pulmonary complications, and promote recovery. Nevertheless, prospective work with validated exposure ascertainment is required.

## Supplementary Material

Supplemental Material

Supplemental Material

STROBE.doc

supplementary_data_S1clean.doc

supplementary data S2.doc

figure Legends.docx

## Data Availability

The datasets used and analysed during the current study are available from the corresponding author on reasonable request.
