# Supplementary figures and images for: Effect of chronic high-altitude exposure on postoperative pulmonary complications: a retrospective cohort study
Source: Ann Med. 2026 Feb 16;58(1):2627063. doi: 10.1080/07853890.2026.2627063 (PMC12912214; doi:10.1080/07853890.2026.2627063)

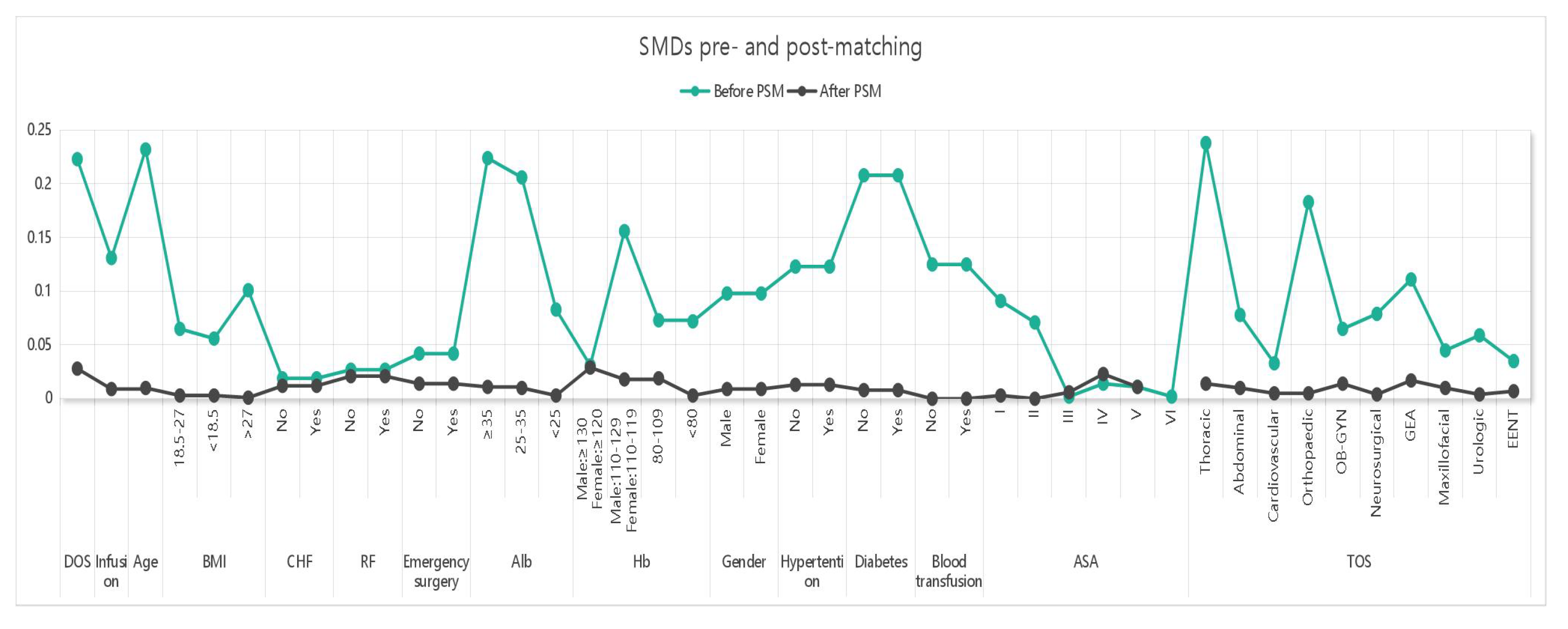

Supplement: Supplemental Material [file IANN_A_2627063_SM8369.tif]
