# Supplementary material for: Effect of chronic high-altitude exposure on postoperative pulmonary complications: a retrospective cohort study
Source: Ann Med. 2026 Feb 16;58(1):2627063. doi: 10.1080/07853890.2026.2627063 (PMC12912214; doi:10.1080/07853890.2026.2627063)
Supplement: supplementary_data_S1clean.doc [file IANN_A_2627063_SM8976.doc]

Data S1

Table S1. The Basic Characteristics and Differences analysis between Chronic High-altitude Exposure group and Non-Chronic High-altitude Exposure group.

| **Variables** | **Total**  **(n = 235127)** | **NCHAE (n = 226562)** | **CHAE (n = 8565)** | **Statistic** | ***P*** |
| --- | --- | --- | --- | --- | --- |
|
| DOS, min, Median (IQR) | 90.00 (55.00, 150.00) | 90.00 (55.00, 150.00) | 110.00 (65.00, 185.00) | Z=-23.65 | **<.001** |
| Intraoperative fluid administration, ml/kg/h, M (Q₁, Q₃) | 8.97 (6.43, 12.80) | 9.00 (6.44, 12.86) | 8.32 (6.04, 11.57) | Z=-14.05 | **<.001** |
| Age, year, Median (IQR) | 49.00 (35.00, 60.00) | 49.00 (36.00, 61.00) | 45.00 (34.00, 55.00) | Z=-19.59 | **<.001** |
| BMI, n (%) |  |  |  | χ²=126.56 | **<.001** |
| 18.5-27 | 206031 (87.63) | 198716 (87.71) | 7315 (85.41) |  |  |
| <18.5 | 9012 (3.83) | 8762 (3.87) | 250 (2.92) |  |  |
| >27 | 20084 (8.54) | 19084 (8.42) | 1000 (11.68) |  |  |
| HF, n(%) |  |  |  | χ²=2.40 | 0.122 |
| No | 232975 (99.08) | 224475 (99.08) | 8500 (99.24) |  |  |
| Yes | 2152 (0.92) | 2087 (0.92) | 65 (0.76) |  |  |
| RF, n(%) |  |  |  | χ²=3.61 | 0.057 |
| No | 234532 (99.75) | 225980 (99.74) | 8552 (99.85) |  |  |
| Yes | 595 (0.25) | 582 (0.26) | 13 (0.15) |  |  |
| Emergency, n(%) |  |  |  | χ²=17.43 | **<.001** |
| No | 223946 (95.24) | 215869 (95.28) | 8077 (94.30) |  |  |
| Yes | 11181 (4.76) | 10693 (4.72) | 488 (5.70) |  |  |
| Alb, g/L, n (%) |  |  |  | χ²=790.23 | **<.001** |
| ≥35 | 212914 (90.55) | 205864 (90.86) | 7050 (82.31) |  |  |
| 25-35 | 21355 (9.08) | 19948 (8.80) | 1407 (16.43) |  |  |
| <25 | 858 (0.36) | 750 (0.33) | 108 (1.26) |  |  |
| Hb, g/L |  |  |  | χ²=243.77 | **<.001** |
| Male:≥130  Female:≥120 | 167953 (71.43) | 161720 (71.38) | 6233 (72.77) |  |  |
| Male:110-129  Female:110-119 | 38974 (16.58) | 37969 (16.76) | 1005 (11.73) |  |  |
| 80-109 | 24070 (10.24) | 22993 (10.15) | 1077 (12.57) |  |  |
| <80 | 4130 (1.76) | 3880 (1.71) | 250 (2.92) |  |  |
| Gender, n (%) |  |  |  | χ²=81.38 | **<.001** |
| Male | 93042 (39.57) | 89252 (39.39) | 3790 (44.25) |  |  |
| Female | 142085 (60.43) | 137310 (60.61) | 4775 (55.75) |  |  |
| Hypertention, n (%) |  |  |  | χ²=95.29 | **<.001** |
| No | 204970 (87.17) | 197207 (87.04) | 7763 (90.64) |  |  |
| Yes | 30157 (12.83) | 29355 (12.96) | 802 (9.36) |  |  |
| Diabetes, n (%) |  |  |  | χ²=176.83 | **<.001** |
| No | 219258 (93.25) | 210968 (93.12) | 8290 (96.79) |  |  |
| Yes | 15869 (6.75) | 15594 (6.88) | 275 (3.21) |  |  |
| Blood transfusion, n (%) |  |  |  | χ²=186.34 | **<.001** |
| No | 217270 (92.41) | 209684 (92.55) | 7586 (88.57) |  |  |
| Yes | 17857 (7.59) | 16878 (7.45) | 979 (11.43) |  |  |
| ASA, n (%) |  |  |  | χ²=62.36 | **<.001** |
| I | 46188 (19.64) | 44784 (19.77) | 1404 (16.39) |  |  |
| II | 163199 (69.41) | 156992 (69.29) | 6207 (72.47) |  |  |
| III | 23248 (9.89) | 22395 (9.88) | 853 (9.96) |  |  |
| IV | 2329 (0.99) | 2232 (0.99) | 97 (1.13) |  |  |
| V | 162 (0.07) | 158 (0.07) | 4 (0.05) |  |  |
| VI | 1 (0.00) | 1 (0.00) | 0 (0.00) |  |  |
| TOS, n (%) |  |  |  | χ²=827.90 | **<.001** |
| Thoracic | 15945 (6.78) | 15695 (6.93) | 250 (2.92) |  |  |
| Abdominal | 58219 (24.76) | 55810 (24.63) | 2409 (28.13) |  |  |
| Cardiovascular | 4069 (1.73) | 3881 (1.71) | 188 (2.19) |  |  |
| Orthopaedic | 24150 (10.27) | 22705 (10.02) | 1445 (16.87) |  |  |
| OB-GYN | 45349 (19.29) | 43897 (19.38) | 1452 (16.95) |  |  |
| Neurosurgical | 12486 (5.31) | 11861 (5.24) | 625 (7.30) |  |  |
| GEA | 34137 (14.52) | 33181 (14.65) | 956 (11.16) |  |  |
| Maxillofacial | 6495 (2.76) | 6312 (2.79) | 183 (2.14) |  |  |
| Urologic | 21365 (9.09) | 20716 (9.14) | 649 (7.58) |  |  |
| EENT | 12912 (5.49) | 12504 (5.52) | 408 (4.76) |  |  |
| Z: Mann-Whitney test, χ²: Chi-square test; M: Median, Q₁: 1st Quartile, Q₃: 3st Quartile; IQR, interquartile range; PSM, Propensity Score Matching; ASA, American Society of Anesthesiologists; BMI, body mass index; Alb,albumin ;CHAE, Chronic high-altitude exposure; NCHAE, Non-Chronic high-altitude exposure; DOS, Duration of surgery; CHF, congestive heart failure; RF, renal failure; Hb, hemoglobin; TOS, type of surgery; OB-GYN, obstetrics and gynecology; GEA, general except Abdominal; EENT, eyes, ears, nose, and throat surgery. | | | | | |

Table S2. Univariate Logistic Regression Results Before PSM

| Variables | β | S.E | Z | *P* | OR (95%CI) |
| --- | --- | --- | --- | --- | --- |
|
| High altitude |  |  |  |  |  |
| No |  |  |  |  | 1.00 (Reference) |
| Yes | 0.20 | 0.05 | 4.18 | **<.001** | 1.22 (1.11 ~ 1.34) |
| BMI, Kg/m^2^ |  |  |  |  |  |
| 18.5-27 |  |  |  |  | 1.00 (Reference) |
| <18.5 | 0.26 | 0.05 | 5.81 | **<.001** | 1.30 (1.19 ~ 1.43) |
| >27 | 0.01 | 0.03 | 0.43 | 0.669 | 1.01 (0.95 ~ 1.09) |
| HF, n(%) |  |  |  |  |  |
| No |  |  |  |  | 1.00 (Reference) |
| Yes | 2.41 | 0.05 | 51.64 | **<.001** | 11.10 (10.13 ~ 12.16) |
| RF |  |  |  |  |  |
| No |  |  |  |  | 1.00 (Reference) |
| Yes | 1.56 | 0.10 | 14.91 | **<.001** | 4.78 (3.89 ~ 5.87) |
| Emergency |  |  |  |  |  |
| No |  |  |  |  | 1.00 (Reference) |
| Yes | 0.51 | 0.04 | 13.68 | **<.001** | 1.67 (1.55 ~ 1.80) |
| Alb, g/L |  |  |  |  |  |
| ≥35 |  |  |  |  | 1.00 (Reference) |
| 25-35 | 1.04 | 0.02 | 41.86 | **<.001** | 2.82 (2.69 ~ 2.96) |
| <25 | 1.83 | 0.08 | 21.62 | **<.001** | 6.24 (5.29 ~ 7.37) |
| Gender |  |  |  |  |  |
| Male |  |  |  |  | 1.00 (Reference) |
| Female | -0.66 | 0.02 | -33.81 | **<.001** | 0.52 (0.50 ~ 0.54) |
| Hypertention |  |  |  |  |  |
| No |  |  |  |  | 1.00 (Reference) |
| Yes | 1.02 | 0.02 | 46.15 | **<.001** | 2.79 (2.67 ~ 2.91) |
| Diabetes |  |  |  |  |  |
| No |  |  |  |  | 1.00 (Reference) |
| Yes | 0.52 | 0.03 | 16.33 | **<.001** | 1.69 (1.58 ~ 1.80) |
| Blood transfusion |  |  |  |  |  |
| No |  |  |  |  | 1.00 (Reference) |
| Yes | 1.76 | 0.02 | 77.72 | **<.001** | 5.81 (5.56 ~ 6.07) |
| DOS, min | 0.01 | 0.00 | 60.71 | **<.001** | 1.01 (1.01 ~ 1.01) |
| Intraoperative fluid administration, ml/kg/h | 0.00 | 0.00 | 0.45 | 0.655 | 1.00 (1.00 ~ 1.00) |
| ASA |  |  |  |  |  |
| I |  |  |  |  | 1.00 (Reference) |
| II | 1.13 | 0.04 | 26.38 | **<.001** | 3.08 (2.83 ~ 3.35) |
| III | 2.40 | 0.05 | 53.13 | **<.001** | 11.03 (10.10 ~ 12.05) |
| IV | 3.90 | 0.06 | 66.44 | **<.001** | 49.50 (44.12 ~ 55.54) |
| V | 3.99 | 0.16 | 24.27 | **<.001** | 53.96 (39.10 ~ 74.47) |
| VI | -4.25 | 43.95 | -0.10 | 0.923 | 0.01 (0.00 ~ 368405469255172448745119693504774144.00) |
| Age, year | 0.05 | 0.00 | 69.06 | **<.001** | 1.05 (1.05 ~ 1.05) |
| TOS |  |  |  |  |  |
| Thoracic |  |  |  |  | 1.00 (Reference) |
| Abdominal | -1.30 | 0.03 | -42.83 | **<.001** | 0.27 (0.26 ~ 0.29) |
| Cardiovascular | 1.04 | 0.04 | 25.64 | **<.001** | 2.82 (2.60 ~ 3.05) |
| Orthopaedic | -1.74 | 0.04 | -38.99 | **<.001** | 0.18 (0.16 ~ 0.19) |
| OB-GYN | -3.65 | 0.07 | -48.81 | **<.001** | 0.03 (0.02 ~ 0.03) |
| Neurosurgical | 0.44 | 0.03 | 14.03 | **<.001** | 1.56 (1.46 ~ 1.66) |
| GEA | -2.36 | 0.05 | -48.10 | **<.001** | 0.09 (0.09 ~ 0.10) |
| Maxillofacial | -3.25 | 0.16 | -20.81 | **<.001** | 0.04 (0.03 ~ 0.05) |
| Urologic | -1.47 | 0.04 | -34.51 | **<.001** | 0.23 (0.21 ~ 0.25) |
| EENT | -4.35 | 0.19 | -22.85 | **<.001** | 0.01 (0.01 ~ 0.02) |
| Hb, g/L |  |  |  |  |  |
| Male:≥130  Female:≥120 |  |  |  |  | 1.00 (Reference) |
| Male:110-129  Female:110-119 | 0.42 | 0.03 | 16.80 | **<.001** | 1.52 (1.45 ~ 1.60) |
| 80-109 | 0.71 | 0.03 | 25.87 | **<.001** | 2.03 (1.92 ~ 2.14) |
| <80 | 0.94 | 0.05 | 17.27 | **<.001** | 2.57 (2.31 ~ 2.86) |
| OR: Odds Ratio, CI: Confidence Interval, IQR, interquartile range; PSM, Propensity Score Matching; ASA, American Society of Anesthesiologists; BMI, body mass index; Alb,albumin ;CHAE, Chronic high-altitude exposure; NCHAE, Non-Chronic high-altitude exposure; DOS, Duration of surgery; CHF, congestive heart failure; RF, renal failure; Hb, hemoglobin; TOS, type of surgery; OB-GYN, obstetrics and gynecology; GEA, general except Abdominal; EENT, eyes, ears, nose, and throat surgery. | | | | | |

Table S3. Patient Baseline Characteristics Before and After Propensity Score Matching.

| **Before PSM** | | | | | **After PSM** | | | |
| --- | --- | --- | --- | --- | --- | --- | --- | --- |
| **Variable** | **Total**  **(n = 235127)** | **NCHAE**  **(n = 226562)** | **CHAE**  **(n = 8565)** | **SMD** | **Total**  **(n = 17128)** | **NCHAE**  **(n = 8564)** | **CHAE (n = 8564)** | **SMD** |
| Age, year, Median (IQR) | 49 (35, 60) | 49 (36, 61) | 45 (34, 55) | **0.232** | 45 (33, 55) | 45 (33, 56) | 45 (34, 55) | 0 |
| Gender, n (%) |  |  |  |  |  |  |  |  |
| Male | 93042 (39.57) | 89252 (39.39) | 3790 (44.25) | 0.098 | 7711 (45.02) | 3922 (45.80) | 3789 (44.24) | 0.031 |
| Female | 142085 (60.43) | 137310 (60.61) | 4775 (55.75) | 0.098 | 9417 (54.98) | 4642 (54.20) | 4775 (55.76) | 0.031 |
| BMI, n (%) |  |  |  |  |  |  |  |  |
| 18.5-27 | 206031 (87.63) | 198716 (87.71) | 7315 (85.41) | 0.065 | 14684 (85.73) | 7370 (86.06) | 7314 (85.40) | 0.019 |
| <18.5 | 9012 (3.83) | 8762 (3.87) | 250 (2.92) | 0.056 | 485 (2.83) | 235 (2.74) | 250 (2.92) | 0.01 |
| >27 | 20084 (8.54) | 19084 (8.42) | 1000 (11.68) | **0.101** | 1959 (11.44) | 959 (11.20) | 1000 (11.68) | 0.015 |
| ASA, n (%) |  |  |  |  |  |  |  |  |
| I | 46188 (19.64) | 44784 (19.77) | 1404 (16.39) | 0.091 | 2823 (16.48) | 1419 (16.57) | 1404 (16.39) | 0.005 |
| II | 163199 (69.41) | 156992 (69.29) | 6207 (72.47) | 0.071 | 12520 (73.1) | 6314 (73.73) | 6206 (72.47) | 0.028 |
| III | 23248 (9.89) | 22395 (9.88) | 853 (9.96) | 0.002 | 1588 (9.27) | 735 (8.58) | 853 (9.96) | 0.046 |
| IV | 2329 (0.99) | 2232 (0.99) | 97 (1.13) | 0.014 | 190 (1.11) | 93 (1.09) | 97 (1.13) | 0.004 |
| V | 162 (0.07) | 158 (0.07) | 4 (0.05) | 0.011 | 7 (0.04) | 3 (0.04) | 4 (0.05) | 0.005 |
| VI | 1 (0) | 1 (0.00) | 0 (0.00) | 0.002 |  |  |  |  |
| Hb, g/L |  |  |  |  |  |  |  |  |
| Male:≥130  Female:≥120 | 167953 (71.43) | 161720 (71.38) | 6233 (72.77) | 0.031 | 12548 (73.26) | 6316 (73.75) | 6232 (72.77) | 0.022 |
| Male:110-129  Female:110-119 | 38974 (16.58) | 37969 (16.76) | 1005 (11.73) | **0.156** | 1920 (11.21) | 915 (10.68) | 1005 (11.74) | 0.033 |
| 80-109 | 24070 (10.24) | 22993 (10.15) | 1077 (12.57) | 0.073 | 2152 (12.56) | 1075 (12.55) | 1077 (12.58) | 0.001 |
| <80 | 4130 (1.76) | 3880 (1.71) | 250 (2.92) | 0.072 | 508 (2.97) | 258 (3.01) | 250 (2.92) | 0.006 |
| Alb, g/L, n (%) |  |  |  |  |  |  |  |  |
| ≥35 | 212914 (90.55) | 205864 (90.86) | 7050 (82.31) | **0.224** | 14199 (82.9) | 7149 (83.48) | 7050 (82.32) | 0.03 |
| 25-35 | 21355 (9.08) | 19948 (8.80) | 1407 (16.43) | **0.206** | 2724 (15.9) | 1317 (15.38) | 1407 (16.43) | 0.028 |
| <25 | 858 (0.36) | 750 (0.33) | 108 (1.26) | **0.083** | 205 (1.2) | 98 (1.14) | 107 (1.25) | 0.009 |
| Hypertention, n (%) |  |  |  |  |  |  |  |  |
| No | 204970 (87.17) | 197207 (87.04) | 7763 (90.64) | **0.123** | 15630 (91.25) | 7868 (91.87) | 7762 (90.64) | 0.042 |
| Yes | 30157 (12.83) | 29355 (12.96) | 802 (9.36) | **0.123** | 1498 (8.75) | 696 (8.13) | 802 (9.36) | 0.042 |
| Diabetes, n (%) |  |  |  |  |  |  |  |  |
| No | 219258 (93.25) | 210968 (93.12) | 8290 (96.79) | **0.208** | 16588 (96.85) | 8299 (96.91) | 8289 (96.79) | 0.007 |
| Yes | 15869 (6.75) | 15594 (6.88) | 275 (3.21) | **0.208** | 540 (3.15) | 265 (3.09) | 275 (3.21) | 0.007 |
| CHF, n (%) |  |  |  |  |  |  |  |  |
| No | 232975 (99.08) | 224475 (99.08) | 8500 (99.24) | 0.019 | 17010 (99.31) | 8511 (99.38) | 8499 (99.24) | 0.016 |
| Yes | 2152 (0.92) | 2087 (0.92) | 65 (0.76) | 0.019 | 118 (0.69) | 53 (0.62) | 65 (0.76) | 0.016 |
| RF (%) |  |  |  |  |  |  |  |  |
| No | 234532 (99.75) | 225980 (99.74) | 8552 (99.85) | 0.027 | 17107 (99.88) | 8556 (99.91) | 8551 (99.85) | 0.015 |
| Yes | 595 (0.25) | 582 (0.26) | 13 (0.15) | 0.027 | 21 (0.12) | 8 (0.09) | 13 (0.15) | 0.015 |
| Emergency surgery, n (%) |  |  |  |  |  |  |  |  |
| No | 223946 (95.24) | 215869 (95.28) | 8077 (94.30) | 0.042 | 16163 (94.37) | 8087 (94.43) | 8076 (94.30) | 0.006 |
| Yes | 11181 (4.76) | 10693 (4.72) | 488 (5.70) | 0.042 | 965 (5.63) | 477 (5.57) | 488 (5.70) | 0.006 |
| DOS, min, Median (IQR) | 90 (55, 150) | 90 (55, 150) | 110 (65, 185) | **0.223** | 110 (65, 180) | 105 (60, 180) | 110 (65, 185) | 0.018 |
| Blood transfusion, n (%) |  |  |  |  |  |  |  |  |
| No | 217270 (92.41) | 209684 (92.55) | 7586 (88.57) | **0.125** | 15232 (88.93) | 7647 (89.29) | 7585 (88.57) | 0.023 |
| Yes | 17857 (7.59) | 16878 (7.45) | 979 (11.43) | **0.125** | 1896 (11.07) | 917 (10.71) | 979 (11.43) | 0.023 |
| TOS, n (%) |  |  |  |  |  |  |  |  |
| Thoracic | 15945 (6.78) | 15695 (6.93) | 250 (2.92) | **0.238** | 504 (2.94) | 254 (2.97) | 250 (2.92) | 0.003 |
| Abdominal | 58219 (24.76) | 55810 (24.63) | 2409 (28.13) | 0.078 | 4730 (27.62) | 2322 (27.11) | 2408 (28.12) | 0.022 |
| Cardiovascular | 4069 (1.73) | 3881 (1.71) | 188 (2.19) | 0.033 | 382 (2.23) | 194 (2.27) | 188 (2.20) | 0.005 |
| Orthopaedic | 24150 (10.27) | 22705 (10.02) | 1445 (16.87) | **0.183** | 2996 (17.49) | 1551 (18.11) | 1445 (16.87) | 0.033 |
| OB-GYN | 45349 (19.29) | 43897 (19.38) | 1452 (16.95) | 0.065 | 2918 (17.04) | 1466 (17.12) | 1452 (16.95) | 0.004 |
| Neurosurgical | 12486 (5.31) | 11861 (5.24) | 625 (7.30) | 0.079 | 1232 (7.19) | 607 (7.09) | 625 (7.30) | 0.008 |
| GEA | 34137 (14.52) | 33181 (14.65) | 956 (11.16) | **0.111** | 1912 (11.16) | 956 (11.16) | 956 (11.16) | 0 |
| Maxillofacial | 6495 (2.76) | 6312 (2.79) | 183 (2.14) | 0.045 | 345 (2.01) | 162 (1.89) | 183 (2.14) | 0.017 |
| Urologic | 21365 (9.09) | 20716 (9.14) | 649 (7.58) | 0.059 | 1283 (7.49) | 634 (7.40) | 649 (7.58) | 0.007 |
| EENT | 12912 (5.49) | 12504 (5.52) | 408 (4.76) | 0.035 | 826 (4.82) | 418 (4.88) | 408 (4.76) | 0.005 |

IQR, interquartile range; PSM, Propensity Score Matching; ASA, American Society of Anesthesiologists; BMI, body mass index; Alb,albumin ;CHAE, Chronic high-altitude exposure; NCHAE, Non-Chronic high-altitude exposure; DOS, Duration of surgery; CHF, congestive heart failure; RF, renal failure; Hb, hemoglobin; TOS, type of surgery; OB-GYN, obstetrics and gynecology; GEA, general except Abdominal; EENT, eyes, ears, nose, and throat surgery.

Table S4 Comparison of Length of Hospital Stay and Postoperative Length of Hospital Stay in Chronic High-altitude Exposure and Non-Chronic High-altitude Exposure Patients.

|  | Before PSM | | | After PSM | | |
| --- | --- | --- | --- | --- | --- | --- |
|  | CHAE | NCHAE | P-Value | CHAE | NCHAE | P-Value |
| HS | 11（7，16） | 8（5，13） | <0.001 | 11 (7, 16) | 9 (5, 14) | <0.001 |
| PO-HS | 6 (3, 9) | 4 (3, 7) | <0.001 | 6 (3, 9) | 5 (3, 8) | <0.001 |

Abbreviation: PSM, Propensity Score Matching; CHAE, Chronic high-altitude exposure; NCHAE, Non-Chronic high-altitude exposure; HS, Hospital Stay; PO-HS, Postoperative Length of Hospital Stay.
