# Supplementary material for: Effect of chronic high-altitude exposure on postoperative pulmonary complications: a retrospective cohort study
Source: Ann Med. 2026 Feb 16;58(1):2627063. doi: 10.1080/07853890.2026.2627063 (PMC12912214; doi:10.1080/07853890.2026.2627063)
Supplement: supplementary data S2.doc [file IANN_A_2627063_SM8974.doc]

**Data S2**

**Sensitivity analysis**

1. **Chronic high-altitude exposure(CHAE) group as individuals whose registered and current residences are both situated at an altitude of ≥ 2500 m above sea level.**

Table S5. Patients Demographics and Baseline Characteristics after Redefine the Exposure Factors.

|  | Before PSM | | |  | After PSM | | |
| --- | --- | --- | --- | --- | --- | --- | --- |
| Variable | NCHAE (n = 223003) | CHAE (n = 12124) | SMD |  | NCHAE (n = 12123) | CHAE (n = 12123) | SMD |
| DOS, Median (IQR) | 90.00 (55.00, 150.00) | 110.00 (65.00, 180.00) | 0.19 |  | 100.00 (60.00, 175.00) | 110.00 (65.00, 180.00) | 0.003 |
| Age, Median (IQR) | 49.00 (36.00, 61.00) | 46.00 (34.00, 56.00) | -0.186 |  | 46.00 (33.00, 56.00) | 46.00 (34.00, 56.00) | 0.006 |
| BMI, n (%) |  |  |  |  |  |  |  |
| 18.5-27 | 195627 (87.72) | 10404 (85.81) | -0.055 |  | 10436 (86.08) | 10403 (85.81) | -0.008 |
| <18.5 | 8667 (3.89) | 345 (2.85) | -0.063 |  | 280 (2.31) | 345 (2.85) | 0.032 |
| >27 | 18709 (8.39) | 1375 (11.34) | 0.093 |  | 1407 (11.61) | 1375 (11.34) | -0.008 |
| CHF, n (%) |  |  |  |  |  |  |  |
| NO | 220950 (99.08) | 12025 (99.18) | 0.012 |  | 12030 (99.23) | 12024 (99.18) | -0.005 |
| YES | 2053 (0.92) | 99 (0.82) | -0.012 |  | 93 (0.77) | 99 (0.82) | 0.005 |
| RF, n (%) |  |  |  |  |  |  |  |
| NO | 222426 (99.74) | 12106 (99.85) | 0.029 |  | 12111 (99.90) | 12105 (99.85) | -0.013 |
| YES | 577 (0.26) | 18 (0.15) | -0.029 |  | 12 (0.10) | 18 (0.15) | 0.013 |
| Emergency surgery, n (%) |  |  |  |  |  |  |  |
| NO | 212504 (95.29) | 11442 (94.37) | -0.04 |  | 11505 (94.90) | 11441 (94.37) | -0.023 |
| YES | 10499 (4.71) | 682 (5.63) | 0.04 |  | 618 (5.10) | 682 (5.63) | 0.023 |
| Alb, n (%) |  |  |  |  |  |  |  |
| ≥35 | 202820 (90.95) | 10094 (83.26) | -0.206 |  | 10189 (84.05) | 10094 (83.26) | -0.021 |
| 25-35 | 19455 (8.72) | 1900 (15.67) | 0.191 |  | 1823 (15.04) | 1900 (15.67) | 0.017 |
| <25 | 728 (0.33) | 130 (1.07) | 0.072 |  | 111 (0.92) | 129 (1.06) | 0.014 |
| Hb |  |  |  |  |  |  |  |
| Male:≥130  Female:≥120 | 159059 (71.33) | 8894 (73.36) | 0.046 |  | 8891 (73.34) | 8893 (73.36) | 0 |
| Male:110-129  Female:110-119 | 37541 (16.83) | 1433 (11.82) | -0.155 |  | 1427 (11.77) | 1433 (11.82) | 0.002 |
| 80-109 | 22607 (10.14) | 1463 (12.07) | 0.059 |  | 1474 (12.16) | 1463 (12.07) | -0.003 |
| <80 | 3796 (1.70) | 334 (2.75) | 0.064 |  | 331 (2.73) | 334 (2.76) | 0.002 |
| Gender, n (%) |  |  |  |  |  |  |  |
| Male | 87730 (39.34) | 5312 (43.81) | 0.09 |  | 5451 (44.96) | 5311 (43.81) | -0.023 |
| Female | 135273 (60.66) | 6812 (56.19) | -0.09 |  | 6672 (55.04) | 6812 (56.19) | 0.023 |
| Hypertention,  n (%) |  |  |  |  |  |  |  |
| No | 194035 (87.01) | 10935 (90.19) | 0.107 |  | 11061 (91.24) | 10934 (90.19) | -0.035 |
| Yes | 28968 (12.99) | 1189 (9.81) | -0.107 |  | 1062 (8.76) | 1189 (9.81) | 0.035 |
| Diabetes, n (%) |  |  |  |  |  |  |  |
| No | 207595 (93.09) | 11663 (96.20) | 0.162 |  | 11698 (96.49) | 11662 (96.20) | -0.016 |
| Yes | 15408 (6.91) | 461 (3.80) | -0.162 |  | 425 (3.51) | 461 (3.80) | 0.016 |
| Blood transfusion, n (%) |  |  |  |  |  |  |  |
| No | 206470 (92.59) | 10800 (89.08) | -0.112 |  | 10833 (89.36) | 10799 (89.08) | -0.009 |
| Yes | 16533 (7.41) | 1324 (10.92) | 0.112 |  | 1290 (10.64) | 1324 (10.92) | 0.009 |
| ASA, n (%) |  |  |  |  |  |  |  |
| I | 44162 (19.80) | 2026 (16.71) | -0.083 |  | 2006 (16.55) | 2026 (16.71) | 0.004 |
| II | 154476 (69.27) | 8723 (71.95) | 0.06 |  | 8853 (73.03) | 8722 (71.95) | -0.024 |
| III | 22020 (9.87) | 1228 (10.13) | 0.008 |  | 1123 (9.26) | 1228 (10.13) | 0.029 |
| IV | 2191 (0.98) | 138 (1.14) | 0.015 |  | 132 (1.09) | 138 (1.14) | 0.005 |
| V | 153 (0.07) | 9 (0.07) | 0.002 |  | 9 (0.07) | 9 (0.07) | 0 |
| VI | 1 (0.00) | 0 (0.00) | -0.002 |  |  |  |  |
| TOS, n (%) |  |  |  |  |  |  |  |
| Thoracic | 15540 (6.97) | 405 (3.34) | -0.202 |  | 391 (3.23) | 405 (3.34) | 0.006 |
| Abdominal | 54868 (24.60) | 3351 (27.64) | 0.068 |  | 3217 (26.54) | 3350 (27.63) | 0.025 |
| Cardiovascular | 3797 (1.70) | 272 (2.24) | 0.037 |  | 285 (2.35) | 272 (2.24) | -0.007 |
| Orthopaedic | 22185 (9.95) | 1965 (16.21) | 0.17 |  | 1983 (16.36) | 1965 (16.21) | -0.004 |
| OB-GYN | 43281 (19.41) | 2068 (17.06) | -0.063 |  | 2146 (17.70) | 2068 (17.06) | -0.017 |
| Neurosurgical | 11589 (5.20) | 897 (7.40) | 0.084 |  | 876 (7.23) | 897 (7.40) | 0.007 |
| GEA | 32710 (14.67) | 1427 (11.77) | -0.09 |  | 1480 (12.21) | 1427 (11.77) | -0.014 |
| Maxillofacial | 6218 (2.79) | 277 (2.28) | -0.034 |  | 253 (2.09) | 277 (2.28) | 0.013 |
| Urologic | 20488 (9.19) | 877 (7.23) | -0.075 |  | 876 (7.23) | 877 (7.23) | 0 |
| EENT | 12327 (5.53) | 585 (4.83) | -0.033 |  | 616 (5.08) | 585 (4.83) | -0.012 |

Abbreviation: IQR, interquartile range; PSM, Propensity Score Matching; ASA, American Society of Anesthesiologists; BMI, body mass index; Alb,albumin ;CHAE, Chronic high-altitude exposure; NCHAE, Non-Chronic high-altitude exposure; DOS, Duration of surgery; CHF, congestive heart failure; RF, renal failure; Hb, haemoglobin; TOS, type of surgery; OB-GYN, obstetrics and gynaecology; GEA, general except Abdominal; EENT, eyes, ears, nose, and throat surgery.

Table S6. Association between Chronic High-altitude Exposure and Postoperative Pulmonary Complications: Crude, Multivariable, and Propensity Score-Matched Analyses Following Chronic High-altitude Exposure Redefinition.

| Analysis | OR (95 % CI) | P-value |
| --- | --- | --- |
| Crude Analysis | 1.21 (1.12 ~ 1.31) | <0.001 |
| Multivariable Analysis | 1.25 (1.14 ~ 1.36) | <0.001 |
| PSM |  |  |
| With matching | 1.18 (1.05 ~ 1.32) | 0.005 |
| Adjusted for PSM | 1.22 (1.08 ~ 1.38) | 0.002 |

OR, odds ratio; PSM, propensity score matching; CI, confidence intervals.

1. **Redefined the observational period for the primary outcome as 15 days post-surgery.**

Table S7. Patients Demographics and Baseline Characteristics After Observational Period for the Primary Outcome as 15 days Post-Surgery.

|  |  | Before PSM |  |  |  | After PSM |  |
| --- | --- | --- | --- | --- | --- | --- | --- |
| Variable | NCHAE (n = 217408) | CHAE (n = 8026) | SMD |  | NCHAE (n = 8026) | CHAE (n = 8026) | SMD |
| DOS, Median (IQR) | 90.00 (55.00, 145.00) | 110.00 (65.00, 175.00) | 0.218 |  | 100.00 (60.00, 170.00) | 110.00 (65.00, 175.00) | 0.019 |
| Age, Median (IQR) | 49.00 (35.00, 60.00) | 45.00 (34.00, 55.00) | -0.226 |  | 45.00 (33.00, 55.00) | 45.00 (34.00, 55.00) | 0 |
| BMI, n (%) |  |  |  |  |  |  |  |
| 18.5-27 | 190811 (87.77) | 6856 (85.42) | -0.066 |  | 6884 (85.77) | 6856 (85.42) | -0.01 |
| <18.5 | 8287 (3.81) | 229 (2.85) | -0.058 |  | 163 (2.03) | 229 (2.85) | 0.049 |
| >27 | 18310 (8.42) | 941 (11.72) | 0.103 |  | 979 (12.20) | 941 (11.72) | -0.015 |
| CHF, n (%) |  |  |  |  |  |  |  |
| No | 215841 (99.28) | 7981 (99.44) | 0.021 |  | 7984 (99.48) | 7981 (99.44) | -0.005 |
| Yes | 1567 (0.72) | 45 (0.56) | -0.021 |  | 42 (0.52) | 45 (0.56) | 0.005 |
| RF (%) |  |  |  |  |  |  |  |
| No | 216911 (99.77) | 8016 (99.88) | 0.029 |  | 8020 (99.93) | 8016 (99.88) | -0.014 |
| Yes | 497 (0.23) | 10 (0.12) | -0.029 |  | 6 (0.07) | 10 (0.12) | 0.014 |
| Emergency surgery  , n (%) |  |  |  |  |  |  |  |
| No | 207286 (95.34) | 7584 (94.49) | -0.037 |  | 7591 (94.58) | 7584 (94.49) | -0.004 |
| Yes | 10122 (4.66) | 442 (5.51) | 0.037 |  | 435 (5.42) | 442 (5.51) | 0.004 |
| Alb, n (%) |  |  |  |  |  |  |  |
| ≥35 | 198586 (91.34) | 6686 (83.30) | -0.216 |  | 6794 (84.65) | 6686 (83.30) | -0.036 |
| 25-35 | 18224 (8.38) | 1251 (15.59) | 0.199 |  | 1142 (14.23) | 1251 (15.59) | 0.037 |
| <25 | 598 (0.28) | 89 (1.11) | 0.08 |  | 90 (1.12) | 89 (1.11) | -0.001 |
| Hb |  |  |  |  |  |  |  |
| Male:≥130  Female:≥120 | 156586 (72.02) | 5872 (73.16) | 0.026 |  | 5918 (73.74) | 5872 (73.16) | -0.013 |
| Male:110-129  Female:110-119 | 35881 (16.50) | 943 (11.75) | -0.148 |  | 915 (11.40) | 943 (11.75) | 0.011 |
| 80-109 | 21404 (9.85) | 983 (12.25) | 0.073 |  | 976 (12.16) | 983 (12.25) | 0.003 |
| <80 | 3537 (1.63) | 228 (2.84) | 0.073 |  | 217 (2.70) | 228 (2.84) | 0.008 |
| Gender, n (%) |  |  |  |  |  |  |  |
| Male | 84177 (38.72) | 3477 (43.32) | 0.093 |  | 3559 (44.34) | 3477 (43.32) | -0.021 |
| Female | 133231 (61.28) | 4549 (56.68) | -0.093 |  | 4467 (55.66) | 4549 (56.68) | 0.021 |
| Hypertention,  n (%) |  |  |  |  |  |  |  |
| No | 190360 (87.56) | 7313 (91.12) | 0.125 |  | 7375 (91.89) | 7313 (91.12) | -0.027 |
| Yes | 27048 (12.44) | 713 (8.88) | -0.125 |  | 651 (8.11) | 713 (8.88) | 0.027 |
| Diabetes, n (%) |  |  |  |  |  |  |  |
| No | 202801 (93.28) | 7769 (96.80) | 0.2 |  | 7759 (96.67) | 7769 (96.80) | 0.007 |
| Yes | 14607 (6.72) | 257 (3.20) | -0.2 |  | 267 (3.33) | 257 (3.20) | -0.007 |
| Blood transfusion, n (%) |  |  |  |  |  |  |  |
| No | 204049 (93.86) | 7238 (90.18) | -0.123 |  | 7270 (90.58) | 7238 (90.18) | -0.013 |
| Yes | 13359 (6.14) | 788 (9.82) | 0.123 |  | 756 (9.42) | 788 (9.82) | 0.013 |
| ASA, n (%) |  |  |  |  |  |  |  |
| I | 44148 (20.31) | 1367 (17.03) | -0.087 |  | 1400 (17.44) | 1367 (17.03) | -0.011 |
| II | 151623 (69.74) | 5874 (73.19) | 0.078 |  | 5926 (73.84) | 5874 (73.19) | -0.015 |
| III | 19966 (9.18) | 712 (8.87) | -0.011 |  | 633 (7.89) | 712 (8.87) | 0.035 |
| IV | 1554 (0.71) | 71 (0.88) | 0.018 |  | 65 (0.81) | 71 (0.88) | 0.008 |
| V | 116 (0.05) | 2 (0.02) | -0.018 |  | 2 (0.02) | 2 (0.02) | 0 |
| VI | 1 (0.00) | 0 (0.00) | -0.002 |  |  |  |  |
| TOS, n (%) |  |  |  |  |  |  |  |
| Thoracic | 15304 (7.04) | 229 (2.85) | -0.251 |  | 193 (2.40) | 229 (2.85) | 0.027 |
| Abdominal | 53531 (24.62) | 2256 (28.11) | 0.078 |  | 2154 (26.84) | 2256 (28.11) | 0.028 |
| Cardiovascular | 2960 (1.36) | 153 (1.91) | 0.04 |  | 143 (1.78) | 153 (1.91) | 0.009 |
| Orthopaedic | 21229 (9.76) | 1328 (16.55) | 0.182 |  | 1427 (17.78) | 1328 (16.55) | -0.033 |
| OB-GYN | 43359 (19.94) | 1427 (17.78) | -0.057 |  | 1448 (18.04) | 1427 (17.78) | -0.007 |
| Neurosurgical | 10109 (4.65) | 495 (6.17) | 0.063 |  | 497 (6.19) | 495 (6.17) | -0.001 |
| GEA | 32562 (14.98) | 930 (11.59) | -0.106 |  | 957 (11.92) | 930 (11.59) | -0.011 |
| Maxillofacial | 6177 (2.84) | 178 (2.22) | -0.042 |  | 162 (2.02) | 178 (2.22) | 0.014 |
| Urologic | 19793 (9.10) | 623 (7.76) | -0.05 |  | 602 (7.50) | 623 (7.76) | 0.01 |
| EENT | 12384 (5.70) | 407 (5.07) | -0.028 |  | 443 (5.52) | 407 (5.07) | -0.02 |

Abbreviation: IQR, interquartile range; PSM, Propensity Score Matching; ASA, American Society of Anesthesiologists; BMI, body mass index; Alb,albumin ;CHAE, Chronic high-altitude exposure; NCHAE, Non-Chronic high-altitude exposure; DOS, Duration of surgery; CHF, congestive heart failure; RF, renal failure; Hb, haemoglobin; TOS, type of surgery; OB-GYN, obstetrics and gynaecology; GEA, general except Abdominal; EENT, eyes, ears, nose, and throat surgery.

Table S8. Association between Chronic High-altitude Exposure and Postoperative Pulmonary Complications in the Crude Analysis, Multivariable Analysis, and Propensity Score Matching Analyses After Observational Period for the Primary Outcome as 15 days Post-Surgery.

| Analysis | OR (95 % CI) | P-value |
| --- | --- | --- |
| Crude Analysis | 1.19 (1.07~ 1.33) | 0.001 |
| Multivariable Analysis | 1.33 (1.18 ~ 1.49) | <0.001 |
| PSM |  |  |
| With matching | 1.26 (1.08 ~ 1.48) | 0.004 |
| Adjusted for PSM | 1.33(1.10 ~ 1.61) | 0.004 |

OR, odds ratio; PSM, propensity score matching; CI, confidence intervals.

1. **Transformed continuous variables in the data into categorical variables.**

Table S9. Patients Demographics and Baseline Characteristics after Transformed Continuous Variables into Categorical Variables.

|  |  | Before PSM |  |  |  | After PSM |  |
| --- | --- | --- | --- | --- | --- | --- | --- |
| Variable | NCHAE (n = 226562) | CHAE (n = 8565) | SMD |  | NCHAE (n = 8563) | CHAE (n = 8563) | SMD |
| BMI, n (%) |  |  |  |  |  |  |  |
| 18.5-27 | 198716 (87.71) | 7315 (85.41) | -0.065 |  | 7309 (85.36) | 7314 (85.41) | 0.002 |
| <18.5 | 8762 (3.87) | 250 (2.92) | -0.056 |  | 203 (2.37) | 250 (2.92) | 0.033 |
| >27.0 | 19084 (8.42) | 1000 (11.68) | 0.101 |  | 1051 (12.27) | 999 (11.67) | -0.019 |
| CHF, n (%) |  |  |  |  |  |  |  |
| No | 224475 (99.08) | 8500 (99.24) | 0.019 |  | 8506 (99.33) | 8498 (99.24) | -0.011 |
| Yes | 2087 (0.92) | 65 (0.76) | -0.019 |  | 57 (0.67) | 65 (0.76) | 0.011 |
| RF (%) |  |  |  |  |  |  |  |
| No | 225980 (99.74) | 8552 (99.85) | 0.027 |  | 8551 (99.86) | 8550 (99.85) | -0.003 |
| Yes | 582 (0.26) | 13 (0.15) | -0.027 |  | 12 (0.14) | 13 (0.15) | 0.003 |
| Emergency surgery  , n (%) |  |  |  |  |  |  |  |
| No | 215869 (95.28) | 8077 (94.30) | -0.042 |  | 8082 (94.38) | 8075 (94.30) | -0.004 |
| Yes | 10693 (4.72) | 488 (5.70) | 0.042 |  | 481 (5.62) | 488 (5.70) | 0.004 |
| Alb, g/L, n (%) |  |  |  |  |  |  |  |
| ≥35 | 205864 (90.86) | 7050 (82.31) | -0.224 |  | 7123 (83.18) | 7050 (82.33) | -0.022 |
| 25-35 | 19948 (8.80) | 1407 (16.43) | 0.206 |  | 1336 (15.60) | 1407 (16.43) | 0.022 |
| <25 | 750 (0.33) | 108 (1.26) | 0.083 |  | 104 (1.21) | 106 (1.24) | 0.002 |
| Hb, g/L |  |  |  |  |  |  |  |
| Male:≥130  Female:≥120 | 161720 (71.38) | 6233 (72.77) | 0.031 |  | 6162 (71.96) | 6233 (72.79) | 0.019 |
| Male:110-129  Female:110-119 | 37969 (16.76) | 1005 (11.73) | -0.156 |  | 1021 (11.92) | 1005 (11.74) | -0.006 |
| 80-109 | 22993 (10.15) | 1077 (12.57) | 0.073 |  | 1139 (13.30) | 1077 (12.58) | -0.022 |
| <80 | 3880 (1.71) | 250 (2.92) | 0.072 |  | 241 (2.81) | 248 (2.90) | 0.005 |
| Gender, n (%) |  |  |  |  |  |  |  |
| Male | 89252 (39.39) | 3790 (44.25) | 0.098 |  | 3830 (44.73) | 3788 (44.24) | -0.01 |
| Female | 137310 (60.61) | 4775 (55.75) | -0.098 |  | 4733 (55.27) | 4775 (55.76) | 0.01 |
| Hypertention,  n (%) |  |  |  |  |  |  |  |
| No | 197207 (87.04) | 7763 (90.64) | 0.123 |  | 7767 (90.70) | 7761 (90.63) | -0.002 |
| Yes | 29355 (12.96) | 802 (9.36) | -0.123 |  | 796 (9.30) | 802 (9.37) | 0.002 |
| Diabetes, n (%) |  |  |  |  |  |  |  |
| No | 210968 (93.12) | 8290 (96.79) | 0.208 |  | 8294 (96.86) | 8288 (96.79) | -0.004 |
| Yes | 15594 (6.88) | 275 (3.21) | -0.208 |  | 269 (3.14) | 275 (3.21) | 0.004 |
| Blood transfusion, n (%) |  |  |  |  |  |  |  |
| No | 209684 (92.55) | 7586 (88.57) | -0.125 |  | 7609 (88.86) | 7586 (88.59) | -0.008 |
| Yes | 16878 (7.45) | 979 (11.43) | 0.125 |  | 954 (11.14) | 977 (11.41) | 0.008 |
| DOS, min, n (%) |  |  |  |  |  |  |  |
| ≤180 | 148537 (65.56) | 4693 (54.79) | -0.216 |  | 4674 (54.58) | 4693 (54.81) | 0.004 |
| 181-240 | 38390 (16.94) | 1703 (19.88) | 0.074 |  | 1720 (20.09) | 1702 (19.88) | -0.005 |
| 241-300 | 17943 (7.92) | 851 (9.94) | 0.067 |  | 884 (10.32) | 851 (9.94) | -0.013 |
| 301-360 | 9993 (4.41) | 543 (6.34) | 0.079 |  | 537 (6.27) | 543 (6.34) | 0.003 |
| 361-420 | 5318 (2.35) | 337 (3.93) | 0.082 |  | 343 (4.01) | 336 (3.92) | -0.004 |
| 421-480 | 2794 (1.23) | 184 (2.15) | 0.063 |  | 179 (2.09) | 184 (2.15) | 0.004 |
| 481-540 | 1499 (0.66) | 107 (1.25) | 0.053 |  | 96 (1.12) | 107 (1.25) | 0.012 |
| 541-600 | 842 (0.37) | 54 (0.63) | 0.033 |  | 61 (0.71) | 54 (0.63) | -0.01 |
| 601-660 | 463 (0.20) | 35 (0.41) | 0.032 |  | 23 (0.27) | 35 (0.41) | 0.022 |
| >660 | 783 (0.35) | 58 (0.68) | 0.04 |  | 46 (0.54) | 58 (0.68) | 0.017 |
| Infusion volume,ml/Kg/h,  Median (IQR), n (%) |  |  |  |  |  |  |  |
| ≤4.0 | 14319 (6.32) | 616 (7.19) | 0.034 |  | 622 (7.26) | 616 (7.19) | -0.003 |
| 4.1-6.0 | 33042 (14.58) | 1501 (17.52) | 0.077 |  | 1531 (17.88) | 1500 (17.52) | -0.01 |
| 6.1-8.0 | 45557 (20.11) | 1915 (22.36) | 0.054 |  | 1938 (22.63) | 1914 (22.35) | -0.007 |
| 8.1-12.0 | 68179 (30.09) | 2577 (30.09) | 0 |  | 2564 (29.94) | 2577 (30.09) | 0.003 |
| 12.1-16.0 | 32857 (14.50) | 1090 (12.73) | -0.053 |  | 1083 (12.65) | 1090 (12.73) | 0.002 |
| 16.1-20.0 | 15892 (7.01) | 437 (5.10) | -0.087 |  | 425 (4.96) | 437 (5.10) | 0.006 |
| >20 | 16716 (7.38) | 429 (5.01) | -0.109 |  | 400 (4.67) | 429 (5.01) | 0.016 |
| ASA, n (%) |  |  |  |  |  |  |  |
| I | 44784 (19.77) | 1404 (16.39) | -0.091 |  | 1383 (16.15) | 1404 (16.40) | 0.007 |
| II | 156992 (69.29) | 6207 (72.47) | 0.071 |  | 6258 (73.08) | 6205 (72.46) | -0.014 |
| III | 22395 (9.88) | 853 (9.96) | 0.002 |  | 829 (9.68) | 853 (9.96) | 0.009 |
| IV | 2232 (0.99) | 97 (1.13) | 0.014 |  | 90 (1.05) | 97 (1.13) | 0.008 |
| V | 158 (0.07) | 4 (0.05) | -0.011 |  | 3 (0.04) | 4 (0.05) | 0.005 |
| VI | 1 (0.00) | 0 (0.00) | -0.002 |  |  |  |  |
| Age, Year, n (%) |  |  |  |  |  |  |  |
| ≤35 | 56504 (24.94) | 2401 (28.03) | 0.069 |  | 2344 (27.37) | 2399 (28.02) | 0.014 |
| 36-45 | 37623 (16.61) | 1998 (23.33) | 0.159 |  | 2020 (23.59) | 1998 (23.33) | -0.006 |
| 46-55 | 54717 (24.15) | 2054 (23.98) | -0.004 |  | 2097 (24.49) | 2054 (23.99) | -0.012 |
| 56-65 | 39899 (17.61) | 1192 (13.92) | -0.107 |  | 1183 (13.82) | 1192 (13.92) | 0.003 |
| 66-75 | 27386 (12.09) | 733 (8.56) | -0.126 |  | 740 (8.64) | 733 (8.56) | -0.003 |
| >75 | 10433 (4.60) | 187 (2.18) | -0.166 |  | 179 (2.09) | 187 (2.18) | 0.006 |
| TOS, n (%) |  |  |  |  |  |  |  |
| Thoracic | 15695 (6.93) | 250 (2.92) | -0.238 |  | 235 (2.74) | 250 (2.92) | 0.01 |
| Abdominal | 55810 (24.63) | 2409 (28.13) | 0.078 |  | 2300 (26.86) | 2409 (28.13) | 0.028 |
| Cardiovascular | 3881 (1.71) | 188 (2.19) | 0.033 |  | 185 (2.16) | 188 (2.20) | 0.002 |
| Orthopaedic | 22705 (10.02) | 1445 (16.87) | 0.183 |  | 1522 (17.77) | 1443 (16.85) | -0.025 |
| OB-GYN | 43897 (19.38) | 1452 (16.95) | -0.065 |  | 1518 (17.73) | 1452 (16.96) | -0.021 |
| Neurosurgical | 11861 (5.24) | 625 (7.30) | 0.079 |  | 582 (6.80) | 625 (7.30) | 0.019 |
| GEA | 33181 (14.65) | 956 (11.16) | -0.111 |  | 946 (11.05) | 956 (11.16) | 0.004 |
| Maxillofacial | 6312 (2.79) | 183 (2.14) | -0.045 |  | 194 (2.27) | 183 (2.14) | -0.009 |
| Urologic | 20716 (9.14) | 649 (7.58) | -0.059 |  | 648 (7.57) | 649 (7.58) | 0 |
| EENT | 12504 (5.52) | 408 (4.76) | -0.035 |  | 433 (5.06) | 408 (4.76) | -0.014 |

Abbreviation: IQR, interquartile range; PSM, Propensity Score Matching; ASA, American Society of Anesthesiologists; BMI, body mass index; Alb,albumin ;CHAE, Chronic high-altitude exposure; NCHAE, Non-Chronic high-altitude exposure; DOS, Duration of surgery; CHF, congestive heart failure; RF, renal failure; Hb, haemoglobin; TOS, type of surgery; OB-GYN, obstetrics and gynaecology; GEA, general except Abdominal; EENT, eyes, ears, nose, and throat surgery.

Table S10. Association between Chronic High-altitude Exposure and Postoperative Pulmonary Complications in the Crude Analysis, Multivariable Analysis, and Propensity Score Matching Analyses after Transformed Continuous Variables into Categorical Variables.

| Analysis | OR (95 % CI) | P-value |
| --- | --- | --- |
| Crude Analysis | 1.22 (1.11~ 1.34) | <0.001 |
| Multivariable Analysis | 1.28 (1.15~ 1.42) | <0.001 |
| PSM |  |  |
| With matching | 1.21 (1.05 ~ 1.39) | 0.01 |
| Adjusted for PSM | 1.21 (1.02 ~ 1.44) | 0.03 |

OR, odds ratio; PSM, propensity score matching; CI, confidence intervals.
