# Supplementary material for: Effect of chronic high-altitude exposure on postoperative pulmonary complications: a retrospective cohort study
Source: Ann Med. 2026 Feb 16;58(1):2627063. doi: 10.1080/07853890.2026.2627063 (PMC12912214; doi:10.1080/07853890.2026.2627063)
Supplement: figure Legends.docx [file IANN_A_2627063_SM8973.docx]

Figure 1. Flow chart of the study.

Figure S1. Propensity score distribution plots.

Figure S2. SMDs pre- and post-matching.

HA, high altitude; PSM, Propensity Score Matching; ASA, American Society of Anesthesiologists; BMI, body mass index; Alb,albumin ;CHAE, Chronic high-altitude exposure; NCHAE, Non-Chronic high-altitude exposure; DOS, Duration of surgery; CHF, congestive heart failure; RF, renal failure; Hb, hemoglobin; TOS, type of surgery; OB-GYN, obstetrics and gynecology; GEA, general except Abdominal; EENT, eyes, ears, nose, and throat surgery.
